# ﻿*Colletotrichumchinense* sp. nov. from *Yuccagloriosa* and *C.quercicola* sp. nov. from *Quercusvariabilis* in China

**DOI:** 10.3897/mycokeys.93.89209

**Published:** 2022-09-26

**Authors:** Cheng-Bin Wang, Ning Jiang, Han Xue, Chun-Gen Piao, Yong Li

**Affiliations:** 1 Key Laboratory of Forest Protection of National Forestry and Grassland Administration, Ecology and Nature Conservation Institute, Chinese Academy of Forestry, Beijing 100091, China Ecology and Nature Conservation Institute, Chinese Academy of Forestry Beijing China

**Keywords:** *Ascomycota*, multigene phylogeny, new species, taxonomy

## Abstract

*Colletotrichum* is an important plant pathogenic genus causing anthracnose on a wide range of host plants. During 2019 and 2021, *Colletotrichum* isolates were obtained during surveys of anthracnose on garden plants in China. Multi-gene phylogenetic analyses of internal transcribed spacer (ITS), glyceraldehyde-3-phosphate dehydrogenase (gapdh), chitin synthase 1 (*chs-1*), actin (act) and beta-tubulin (tub2) sequences coupled with morphological evidence support the introduction of two novel species namely *Colletotrichumchinense* sp. nov. from *Yuccagloriosa* in Beijing and *C.quercicola* sp. nov. from *Quercusvariabilis* in Shaanxi Province. Phylogenetic inference revealed that two isolates of *C.chinense* belonged to the agaves species complex and were closely related to *C.agaves*, and differed from the other species within this species complex by shorter conidia and the host association. Molecular identification showed that two isolates of *C.quercicola* formed a highly supported lineage close to *C.tanaceti* in the destructivum species complex, which could be distinguished from *C.tanaceti* by straighter conidia. In pathogenicity tests, yellow spots and orange conidial masses displayed on the inoculated *Y.gloriosa* leaves and brown spots appeared on the inoculated *Q.variabilis* leaves. In addition, *C.chinense* and *C.quercicola* were re-isolated from spots of the tested leaves of *Y.gloriosa* and *Q.variabilis*.

## ﻿Introduction

The genus *Colletotrichum* (*Glomerellaceae*, *Glomerellales*, *Sordariomycetes*) is represented by its type species *Colletotrichumlineola* (Corda 1831; Damm et al. 2009; Hyde et al. 2020). The sexual morph of *Colletotrichum*, characterized by solitary or gregarious ascomata, 8-spored asci, and one-celled hyaline ascospores, was previously known as the genera *Gnomoniopsis* and *Glomerella* (Stoneman 1898; von Schrenk and Spaulding 1903; Marin-Felix et al. 2017). The asexual morph is characterized by acervular conidiomata, often with setae, producing cylindrical or crescent-shaped conidia, and by the formation of appressoria (Sutton 1992; Marin-Felix et al. 2017). With the implementation of “one fungus one name” nomenclature, *Colletotrichum* has been chosen to represent this genus based on priority (Réblová et al. 2016).

Previously, species of *Colletotrichum* were distinguished based on host range and a suite of morphological characteristics, especially the size and shape of conidia, appressoria, and sporulating structures (von Arx 1957; Cai et al. 2009; Hyde et al. 2009a, b). However, many taxonomic problems arose, due to few reliable and often variable morphological characters among species, and uncertain or broad host relationships (Cai et al. 2009; Hyde et al. 2009a; Cannon et al. 2012; Liu et al. 2016). Thus, many species are required taxonomic revision in order to clarify their taxonomic placement (Weir et al. 2012; Damm et al. 2014; Liu et al. 2022).

To establish a stable and natural classification system, Cai et al. (2009) recommended using a polyphasic approach, emphasizing multi-locus phylogeny in conjunction with morphology, geographical and ecological information to characterize and differentiate *Colletotrichum* species. Subsequently, many *Colletotrichum* species had been successfully identified and epitypified, resulting in a much better understanding of phylogenetic relationships of this genus (Weir et al. 2012; Damm et al. 2014). Currently, more than 1000 *Colletotrichum* epithets are listed in Index Fungorum (http://www.indexfungorum.org), and at least 303 species, grouped in 16 species complexes and some singleton species (Mu et al. 2021; Alizadeh et al. 2022; Liu et al. 2022; Zheng et al. 2022).

Many species of *Colletotrichum* have been identified as plant pathogens causing anthracnose on a wide range of hosts, especially in subtropical and tropical regions, leading to significant economic losses (Hyde et al. 2009a; Cannon et al. 2012; Lima et al. 2013). In addition, *Colletotrichum* species may occur as endophytes, saprobes, or opportunistic human pathogens, sometimes as latent plant pathogens, which may switch to a pathogenic lifestyle depending on the host plant, *Colletotrichum* species, and environmental conditions. (Huang et al. 2013; Rai and Agarkar 2014; Crous et al. 2016a; De Silva et al. 2017).

In the present study, by using a nucleotide basic local alignment search tool (BLASTn) analysis (Boratyn et al. 2013) of the ITS sequences, four *Colletotrichum* isolates from *Yuccagloriosa* and *Quercusvariabilis* showed highest similarity lower than 98% with species in the agaves and destructivum species complex, respectively. The agaves species complex, represented by *Colletotrichumagaves* and four closely related species, occupies a monophyletic clade within this genus (Bhunjun et al. 2021; Talhinhas and Baroncelli 2021). The destructivum species complex is a monophyletic group of *C.destructivum* and 19 closely related species that are mainly plant pathogens (Damm et al. 2014; Bhunjun et al. 2021; Talhinhas and Baroncelli 2021). Members of this species complex are serious economic pathogens, such as *C.destructivum*, *C.lentis* and *C.higginsianum* (Damm et al. 2014; Bhadauria et al. 2019; Khodaei et al. 2019). They are characterized by conidia that are slightly curved due to their unilaterally tapering ends and by the small inconspicuous acervuli with rather effuse growth that are sometimes difficult to spot on the host plants (Damm et al. 2014).

Recently, we investigated the phylogenetic diversity of *Colletotrichum* species associated with anthracnose on garden plants in China. Four novel isolates were collected from *Y.gloriosa* and *Q.variabilis* in Beijing and Shaanxi, respectively. The aim of this study was to identify these isolates based on phylogenetic data and morphology and to confirm their pathogenicity.

## ﻿Materials and methods

### ﻿Sampling and fungal isolation

From 2019 to 2021, symptomatic leaves of garden plants were collected in China. Specimens were transferred to the laboratory in paper bags and stored at 4 °C until further processing. The surface of diseased leaves were sterilized with 70% ethanol and 2% NaClO for 1 min, rinsed three times with sterile water, and then samples were cut into 0.4 × 0.4 cm small pieces excised from the margins of foliar lesions, and placed on potato dextrose agar (PDA; potato extract 20 g, dextrose 20 g, agar 20 g, 1 L distilled water) plates at 25 °C in the dark. After 2–3 days, single colonies growing from the diseased tissue were transferred to new PDA plates. Single-spore cultures were obtained from the pure colonies and examined morphologically. The cultures were deposited in the China Forestry Culture Collection Center (CFCC; http://cfcc.caf.ac.cn/), and the specimens in the herbarium of the Chinese Academy of Forestry (CAF; http://museum.caf.ac.cn/).

### ﻿Morphological and culture characterisation

Agar plugs (6 mm in diameter) were taken from the edge of actively growing cultures on PDA and transferred in triplicate on PDA, synthetic low-nutrient agar (SNA; Nirenberg 1976), and malt extract agar (MEA; malt extract 20 g, agar 20 g, yeast extract 2 g, sucrose 5 g, sterile deionized water 1 L) incubated in the dark at 25 °C. After 7 days, the colony characteristics, colony diameters, and pigment production on the three media were noted. Appressoria were observed on slide cultures according to Weir et al. (2012). Moreover, the shape, color and size of conidia, conidiophores, setae, conidiogenous cells and appressoria were measured and captured at least 20 measurements using a Nikon Eclipse 80i compound microscope with differential interference contrast optics.

### ﻿DNA extraction, PCR amplification and sequencing

Total genomic DNA was extracted from fungal mycelia using a CTAB DNA extraction protocol (Doyle and Doyle 1990). The internal transcribed spacer (ITS), glyceraldehyde-3-phosphate dehydrogenase (*gapdh*), chitin synthase 1 (*chs-1*), actin (*act*) and beta-tubulin (tub2) genes were amplified and sequenced using the primer pairs ITS1/ITS4 (White et al. 1990), GDF1/GDR1 (Guerber et al. 2003), CHS-79F/CHS-345R (Carbone and Kohn 1999), ACT-512F/ACT-783R (Carbone and Kohn 1999) and T1/Bt2b (Glass and Donaldson 1995; O’Donnell and Cigelnik 1997), respectively. PCR was performed in 20 μL reaction mixtures containing 10 μL 2× Taq polymerase (Tiangen, China), which contains a premix of Taq DNA polymerase (0.1 U), dNTPs (0.5 mM), MgCl_2_ (3 mM) Tris-HCl (20 mM), KCl (100 mM) and the appropriate buffer system, 7 μL RNase-free water, 1 μL of each primer (0.5 µM) and 1 μL of DNA template (20 ng/μl). The PCR conditions were as follows: initial heat treatment of 5 min at 94 °C, followed by 35 cycles of 30 sec at 94 °C, 30 s at 54 °C (ITS), 60 °C (*gapdh*), 59 °C (*chs-1*), 58 °C (*act*) or 55 °C (*tub2*), and 1 min at 72 °C, and a final elongation period of 7 min at 72 °C. Amplicons were purified and sequenced by ABI3730XL Gene Analyzer at the Shanghai Invitrogen Biological Technology Company Limited (Beijing, China).

### ﻿Phylogenetic analyses

Newly generated sequences from the four isolates in this study were assembled using SeqMan v. 7.1.0, and the closest match using BLASTn analyses. Reference *Colletotrichum* sequences (Table 1) were downloaded from GenBank, based on recent publication (Liu et al. 2022). Multiple sequences were aligned using the MAFFT v.7.110 online programme (http://mafft.cbrc.jp/alignment/server/, Katoh et al. 2019) by default settings, and adjusted manually in MEGA v.7.0 (Kumar et al. 2016). The best-fit nucleotide substitution models for each gene were selected using jModelTest v. 2.1.7 (Darriba et al. 2012) under the Akaike information criteria (AIC).

**Table 1.** *Colletotrichum* spp. used for phylogenetic analyses in the study.

Species name	Accession no.^a^	GenBank Accession No.				
		ITS	*Gapdh*	*chs-1*	*act*	*tub2*
*agaves*	CBS 118190*	DQ286221	NA	NA	NA	NA
*C.agaves*	LC0947	MZ595831	MZ664053	MZ799266	MZ664129	MZ673955
*C.americae-borealis*	ATCC 11869	KM105223	KM105578	KM105293	KM105433	KM105503
*C.americae-borealis*	CBS 136232*	KM105224	KM105579	KM105294	KM105434	KM105504
*C.antirrhinicola*	CBS 102189*	KM105180	KM105531	KM105250	KM105390	KM105460
*C.atractylicola*	SAUCC 1307*	KR149280	KR259334	KR259333	KR132243	KU058178
*C.atractylicola*	SAUCC 130801	KU289192	KU289207	KU289202	KU289197	KU289212
*C.boninense*	CBS 123755*	JQ005153	JQ005240	JQ005327	JQ005501	JQ005588
*C.brasiliense*	CBS 128501*	JQ005235	JQ005322	JQ005409	JQ005583	JQ005669
*C.bryoniicola*	CBS 109849*	KM105181	KM105532	KM105251	KM105391	KM105461
***C.chinense***	CFCC 57501*	**ON692808**	**ON755050**	**ON755046**	**ON755042**	**ON755054**
***C.chinense***	**CFCC 57502**	**ON692809**	**ON755051**	**ON755047**	**ON755043**	**ON755055**
*C.destructivum*	CBS 114801	KM105219	KM105574	KM105289	KM105429	KM105499
*C.destructivum*	CBS 157.83	KM105215	KM105570	KM105285	KM105425	KM105495
*C.destructivum*	IMI 387103	KM105221	KM105576	KM105291	KM105431	KM105501
*C.destructivum*	CBS 136228*	KM105207	KM105561	KM105277	KM105417	KM105487
*C.euphorbiae*	CBS 134725*	KF777146	KF777131	KF777128	KF777125	KF777247
*C.fuscum*	CBS 133701*	KM105174	KM105524	KM105244	KM105384	KM105454
*C.fuscum*	CBS 133702	KM105178	KM105528	KM105248	KM105388	KM105458
*C.fuscum*	CBS 133703	KM105175	KM105525	KM105245	KM105385	KM105455
*C.fusiforme*	MFLUCC 12-0437	KT290266	KT290255	KT290253	KT290251	KT290256
*C.higginsianum*	CPC 19379*	KM105184	KM105535	KM105254	KM105394	KM105464
*C.higginsianum*	CPC 19364	KM105185	KM105537	KM105255	KM105395	KM105465
*C.higginsianum*	CPC 19369	KM105188	KM105540	KM105258	KM105398	KM105468
*C.higginsianum*	CPC 19394	KM105193	KM105546	KM105263	KM105403	KM105473
*C.ledebouriae*	CBS 141284*	KX228254	NA	NA	KX228357	NA
*C.lentis*	CBS 127604*	JQ005766	KM105597	JQ005787	JQ005829	JQ005850
*C.lentis*	CBS 127605	KM105241	KM105598	KM105311	KM105451	KM105521
*C.lini*	CBS 172.51*	JQ005765	KM105581	JQ005786	JQ005828	JQ005849
*C.lini*	CBS 136856	KM105233	KM105589	KM105303	KM105443	KM105513
*C.lini*	CBS 130828	KM105234	KM105590	KM105304	KM105444	KM105514
*C.neorubicola*	CCR144*	MK529906	MK547520	MK547526	MK547523	MN186400
*C.neorubicola*	CCR145	MK529908	MK547521	MK547527	MK547524	MN186401
*C.neorubicola*	CCR146	MK529907	MK547522	MK547528	MK547525	MN186402
*C.neosansevieriae*	CBS 139918*	KR476747	KR476791	NA	KR476790	KR476797
*C.ocimi*	CBS 298.94*	KM105222	KM105577	KM105292	KM105432	KM105502
*C.panacicola*	C08048	GU935867	GU935847	NA	GU944757	NA
*C.panacicola*	C08061	GU935868	GU935848	NA	GU935791	NA
*C.panacicola*	C08087	GU935869	GU935849	NA	GU944758	NA
*C.pisicola*	CBS 724.97 *	KM105172	KM105522	KM105242	KM105382	KM105452
*C.pleopeltidis*	CBS 147082*	MW883412	NA	MW890035	MW890024	NA
***C.quercicola***	CFCC 54457*	**ON692810**	**ON755052**	**ON755048**	**ON755044**	**ON755056**
***C.quercicola***	**CFCC 57507**	**ON692811**	**ON755053**	**ON755049**	**ON755045**	**ON755057**
*C.sansevieriae*	MAFF 239721*	LC179806	LC180130	LC180129	LC180127	LC180128
*C.sansevieriae*	BTGN2	MN386823	MN386911	NA	NA	MN386867
*C.shisoi*	JCM 31818*	MH660930	MH660931	MH660929	MH660928	MH660932
*C.shisoi*	MAFF 240106	MH660936	MH660935	MH660934	MH660933	MH660937
*C.tabacum*	CBS 124249	KM105206	KM105560	KM105276	KM105416	KM105486
*C.tabacum*	CBS 161.53	JQ005763	KM105559	JQ005784	JQ005826	JQ005847
*C.tabacum*	CPC 18945*	KM105204	KM105557	KM105274	KM105414	KM105484
*C.tanaceti*	BRIP 57316	JX218230	JX218245	JX259270	JX218240	JX218235
*C.tanaceti*	CBS 132693	JX218228	JX218243	JX259268	JX218238	JX218233
*C.tanaceti*	CBS 132818	JX218229	JX218244	JX259269	JX218239	JX218234
*C.truncatum*	IMI 135524	GU227874	GU228266	GU228364	GU227972	GU228168
*C.utrechtense*	CBS 130243*	KM105201	KM105554	KM105271	KM105411	KM105481
*C.utrechtense*	CBS 135827	KM105202	KM105555	KM105272	KM105412	KM105482
*C.utrechtense*	CBS 135828	KM105203	KM105556	KM105273	KM105413	KM105483
*C.vignae*	CBS 501.97*	KM105183	KM105534	KM105253	KM105393	KM105463
*C.vignae*	CPC 19383	KM105182	KM105533	KM105252	KM105392	KM105462

Notes: NA, not applicable. * ex-type strains. ^a^ATCC: American Type Culture Collection, Virginia, USA; BRIP: Plant Pathology Herbarium, Department of Primary Industries, Queensland, Australia; CBS: Culture collection of the Centraalbureau voor Schimmelcultures, Fungal Biodiversity Centre, Utrecht, The Netherlands; CFCC: China Forestry Culture Collection Center, Beijing, China; CPC: Culture collection of Pedro Crous, housed at CBS; LC: the LC Culture Collection (a personal culture collection of Lei Cai, housed in the Institute of Microbiology, Chinese Academy of Sciences); IMI: Culture collection of CABI Europe UK Centre, Egham, UK; JCM: Japan Collection of Microorganisms, RIKEN Bioresource Center, Tsukuba, Japan; MAFF: MAFF Genbank Project, Ministry of Agriculture, Forestry and Fisheries, Tsukuba, Japan; MFLU: Mae Fah Luang University Culture Collection, Thailand; SAUCC: Department of Plant Pathology, College of Plant Protection, Shenyang Agricultural University, China.

Phylogenetic analyses using Maximum Likelihood (ML) and Bayesian Inference (BI) were performed. ML analyses were constructed on the RAxML-HPC BlackBox 8.2.10 (Stamatakis 2014) using the GTR+GAMMA model with 1000 bootstrap replicates. BI analyses were also performed using a Markov Chain Monte Carlo (MCMC) algorithm in MrBayes v. 3.2.6 (Ronquist et al. 2012). The analyses were conducted by running 5,000,000 generations in two independent runs and sampling every 100^th^ generations. The first 25% of the trees of MCMC sampling were discarded as burn-in and posterior probabilities (PP) were determined from the remaining trees. The results were visualized in FigTree 1.4 (http://tree.bio.ed.ac.uk/software/figtree) and edited with Adobe Illustrator CS6.0.

### ﻿Pathogenicity test

The pathogenicity of two *Colletotrichum* isolates was assessed on detached healthy *Y.gloriosa* and *Q.variabilis* plants in the greenhouse. Leaves were washed in running distilled water, surface-sterilized in 70% ethanol and 2% NaClO for 1 min, then rinsed in sterile distilled water. Spores were harvested from two-week-old PDA plates with 10 ml of sterilized water with spore suspension filtered through two layers of cheesecloth to eliminate debris and mycelium. The conidial suspension was adjusted to a final inoculum concentration of 1 × 10^6^–10^7^ conidia/mL with sterile deionized water. Then 10 µL of conidial suspension was placed in the middle portion of the leaves, and inoculated sterile water in the additional leaves served as control. Each treatment had three replicates (three leaves), and the experiment was carried out twice. The inoculated leaves were placed in transparent plastic bags at 25 °C and over 90% humidity in the dark for 14 days. After appearance of symptoms, fungus isolates were re-isolated from the infected leaves and identified based on the morphological and phylogenetic analyses to fulfill Koch’s postulates.

## ﻿Results

### ﻿Phylogenetic analyses

Closest matches in BLASTn searches with the ITS sequences, these isolates were preliminarily identified to be in the agaves and destructivum species complexes. Further, phylogenetic trees were constructed based on combined loci of ITS, *gapdh*, *act*, *chs-1* and *tub2* sequences to identify these isolates to species level.

**Figure 1.**
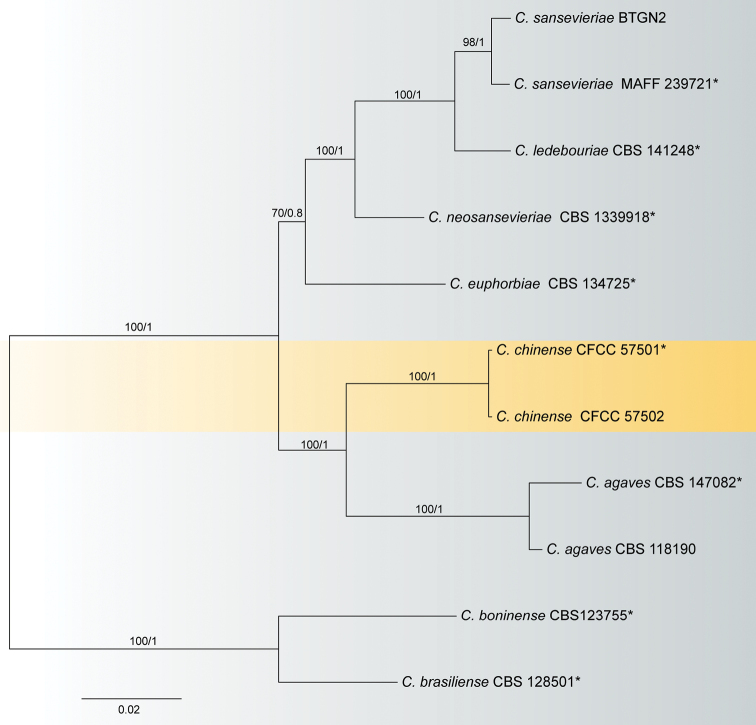
Phylogenetic tree obtained by Maximum likelihood analyses using the combined ITS, *gapdh*, *chs-1*, *act* and *tub2* sequence alignments of the agaves species complex. Numbers above the branches indicate ML bootstraps (left, MLBS ≥ 50%) and Bayesian Posterior Probabilities (right, BPP ≥ 0.7). The tree is rooted with *C.boninense* (CBS 123755, ex-type) and *C.brasiliense* (CBS 128501, ex-type).

For the agaves species complex, DNA sequences of five genes were obtained from two isolates from *Y.gloriosa* in this study, with seven reference strains of the agaves species complex, and *C.boninense* (CBS 123755, ex-type) and *C.brasiliense* (CBS 128501, ex-type) as the outgroup taxa. A total of 1649 characters including alignment gaps (578 for ITS, 94 for *gapdh*, 232 for *chs-1*, 240 for *act* and 505 for *tub2*) were included in the phylogenetic analyses. Of these characters, 1271 were constant, 162 were variable and parsimony-uninformative, and 216 were parsimony-informative. The resulting ML and BI trees had similar topologies; the ML tree (Fig. 1) was selected to represent the phylogeny with ML/BI support values. Two isolates (CFCC 57501 and CFCC 57502) formed a close clade to *C.agaves* (Fig. 1).

**Figure 2.**
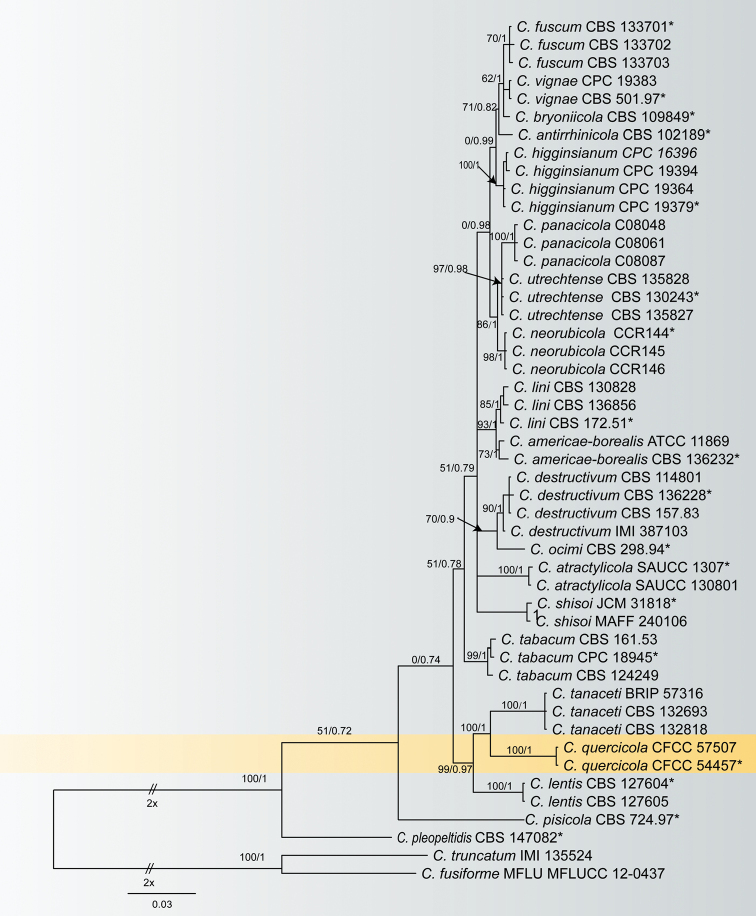
Phylogenetic tree obtained by Maximum likelihood analyses using the combined ITS, *gapdh*, *chs-1*, *act* and *tub2* sequence alignments of the destructivum species complex. Numbers above the branches indicate ML bootstraps (left, MLBS ≥ 50%) and Bayesian Posterior Probabilities (right, BPP ≥ 0.7). The tree is rooted with *C.fusiforme* (MFLU 12-0437CC) and *C.truncatum* (IMI 135524).

For the destructivum species complex, DNA sequences of five genes were obtained from two isolates from *Q.variabilis* in this study, and 44 reference strains of the destructivum species complex, and *C.truncatum* (IMI 135524) and *C.fusiforme* (MFLUCC 12-0437) as the outgroup taxa. A total of 1875 characters including gaps (560 for ITS, 236 for *gapdh*, 280 for *chs-1*, 274 for *act* and 525 for *tub2*) were obtained in the phylogenetic analyses. Of these characters, 1292 were constant, 177 were variable and parsimony-uninformative, and 406 were parsimony-informative. The resulting ML and BI trees had similar topologies; the ML tree (Fig. 2) was selected to represent the phylogeny with ML/BI support values. Two new isolates (CFCC 54457 and CFCC 57507) formed a sister clade to *C.tanaceti* (Fig. 2).

### ﻿Taxonomy

#### ﻿ Colletotrichum chinense

Taxon classificationFungiGlomerellalesGlomerellaceae

Ning Jiang & C.B. Wang sp. nov.

EE9EFA96-FCFB-51CA-AFF1-0D7D517A7F62

Fig. 3

##### Etymology.

Referring to the country, where the species was first collected.

##### Description.

***Sexual morph*** not observed. ***Asexual morph*** developed on PDA. ***Setae*** and ***chlamydospores*** not observed. ***Conidiomata*** acervular, abundant, pulvinate, 200–500 μm diam. ***Conidiophores*** smooth-walled, unbranched, septate, sometimes constricted at the septa, hyaline, up to 40 µm long. ***Conidiogenous cells*** 6.5–19.5 × 3–8 µm (*x*– = 12.7 ± 2.7 × 5.3 ± 1.3 µm, n = 20), subglobose to ampulliform, smooth-walled, hyaline. ***Conidia*** 9.5–25.5 × 3.5–8.5 µm (*x*– = 14.8 ± 1.8 × 6 ± 1 μm, n = 50), L/W ratio = 2–2.7, cylindrical, obtuse at the apex, smooth-walled, hyaline, contents granular. ***Appressoria*** not observed.

##### Culture characters.

Colonies on PDA, flat, with an entire margin, with sparse aerial mycelium, covered with orange conidial masses, reaching 23–25 mm diam in 7 days at 25 °C. Colonies on MEA, flat, with no aerial mycelium, covered with slimy conidial masses, reaching 15–20 diam in 7 days at 25 °C. Colonies on SNA flat, sparse white hyphae, with an entire margin, reaching 12–15 diam in 7 days at 25 °C.

##### Specimens examined.

China, Beijing City, isolated from leaf spot of *Yuccagloriosa* L., *Cheng-Bin Wang*, 15 August 2020 (holotype CAF800056; ex-type living culture: CFCC 57501); *Ibid* (living culture: CFCC 57502).

##### Notes.

*Colletotrichumbeeveri* of the boninense species complex and *C.tofieldiae* of the spaethianum species complex have been reported from *Yucca* before the present study (Liu et al. 2022). *Colletotrichumchinense* from the present study is similar to *C.beeveri* in the conidial shape, but differs in conidial size (9.5–25.5 × 3.5–8.5 µm in PDA vs. 12.5–15.5 × 5.5–6.5 µm in SNA) (Damm et al. 2012). In addition, *C.tofieldiae* differs from *C.chinense* by the falcate conidia (Damm et al. 2009). Based on phylogenetic analyses using multi-locus sequences (ITS, *gapdh*, *chs-1*, *act* and *tub2*), *C.chinense* formed a sister clade to *C.agaves* in the agaves species complex. The sequence identities between *C.chinense*CFCC 57501 and *C.agaves* LC0947 (21/578 ITS, 6/94 *gapdh*, 6/232 *chs-1*, 19/240 *act* and 26/505 *tub2*), *C.euphorbiae*CBS 134725 (31/578 ITS, 8/94 *gapdh*, 7/232 *chs-1*, 35/240 *act* and 32/505 *tub2*), *C.ledebouriae*CBS 141284 (29/578 ITS, 30/240 *act*), *C.neosansevieriae*CBS 139918 (28/578 ITS, 6/94 *gapdh*, 28/240 *act* and 27/416 *tub2*) and *C.sansevieriae*MAFF 239721 (29/578 ITS, 5/94 *gapdh*, 9/232 *chs-1*, 31/240 *act* and 44/505 *tub2*). (Nakamura et al. 2006; Crous et al. 2013, 2015, 2016b; Liu et al. 2022) The *chs-1* sequence of *C.neosansevieriae*CBS 139918 and the *gapdh*, *chs-1* and *tub2* sequences of *C.ledebouriae*CBS 141284 were missing. Morphologically, the conidia size of *C.chinense* are shorter than other species (Table 2).

**Table 2.** Morphological comparison of species in the agaves species complex.

Species	Type	Media for Conidia morph	Hosts	Distribution	Conidia (µm)	Appressoria (µm)	Reference
*C.agaves*	Epitype	PDA	*Agave* spp.	Mexico; USA; Netherlands	(17.5–)19.0–30.5(–33) × 5–8(–9.5) on	Not observed	Farr et al. (2006)
***C.chinense***	**Holotype**	**PDA**	***Yuccagloriosa***	**China**	**(9.5–)12.5–16.5(–25.5) × (3.5–)6.0–7.0(–8.5)**	**Not observed**	**This study**
*C.euphorbiae*	Holotype	SNA	*Euphorbia* sp	South Africa	(17–)23–28(–28.5) × (6–)6.5–7	(6.5–)8.5–14.5(–20.5) × (5.5–)6–10.5(–16)	Crous et al. (2013)
*C.ledebouriae*	Holotype	PNA	*Ledebouriafloribunda*	South Africa	(15–)17–21(–22) × (5–)6	Not observed	Crous et al. (2016)
*C.neosansevieriae*	Holotype	SNA	*Sansevieriatrifasciata*	South Africa	(16–)18–22(–25) × (4–)5–6	Not observed	Crous et al. (2015)
*C.sansevieriae*	Holotype	PDA	*Sansevieria* spp.	Asia; Australia; USA	12.5–(18.4)–32.5 × 3.8–(6.4)–8.8 PDA	6.3–(7.7)–8.8 × 6.3–(7.3)–7.5	Nakamura et al. (2006)

**Figure 3.**
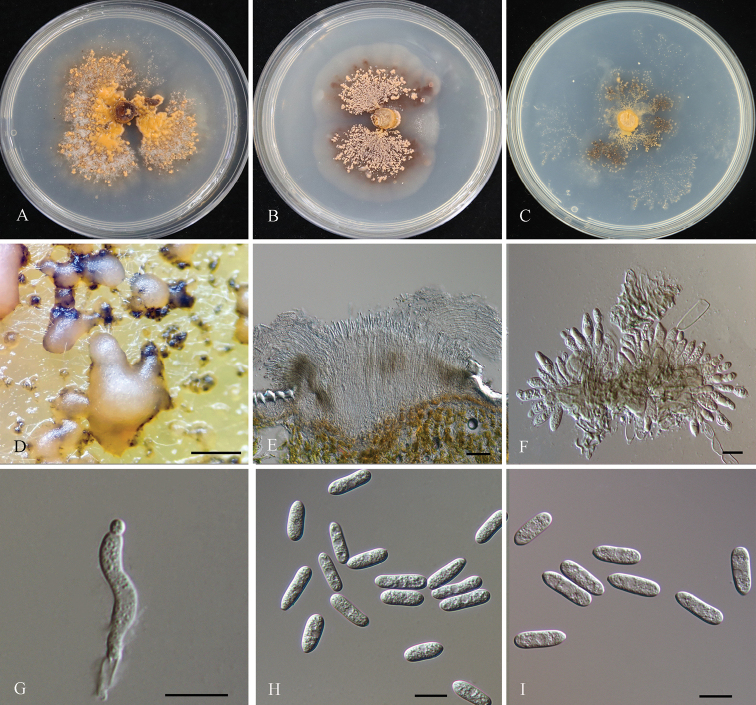
*Colletotrichumchinense* (CFCC 57501; ex-type) **A** colony on PDA**B** colony on MEA**C** colony on SNA**D** conidiomata formed in PDA**E** conidiophores from the host **F, G** conidiophores **H, I** conidia **F–I** from PDA. Scale bars: 200 µm (**D**); 50 µm (**E**); 20 µm (**F, G**); 10 µm (**H, I**).

#### ﻿ Colletotrichum quercicola

Taxon classificationFungiGlomerellalesGlomerellaceae

Ning Jiang & C.B. Wang sp. nov.

501CB79A-1ECF-58CD-9D64-E6256D0FBB2A

Fig. 4

##### Etymology.

Referring to the host genus, *Quercus*.

##### Description.

***Sexual morph*** not observed. ***Asexual morph*** developed on PDA. ***Chlamydospores*** not observed. ***Conidiomata*** acervular, abundant, globose to pulvinate, 200–400 μm diam. ***Conidiophores***, hyaline, branched, smooth-walled, up to 50 μm long. *Setae* medium brown, smooth-walled, 60–145 μm long, 1–3-septate. ***Conidiogenous cells*** 6–18 × 3–7 µm (*x*– = 7.9 ± 3.6 × 4 ± 1.2 µm, n = 20), hyaline, smooth-walled, cylindrical to elongate ampulliform. ***Conidia*** 14.5–23 × 3–5 µm (*x*– = 17 ± 1.7 × 3.9 ± 0.5 μm, n = 50), L/W ratio =4–5, hyaline, smooth-walled, fusiform, straight to slightly curved with both ends rounded or one end round and the other truncate. ***Appressoria*** 6–11 × 4–8 µm (*x*– = 8.4 ± 1.4× 5 ± 1 μm, n = 50), L/W ratio = 1.5–2, single, medium brown, smooth-walled, subglobose, ovate to broadly elliptical in outline.

##### Culture characters.

Colonies on PDA flat, with moderate aerial mycelium, margin white to light gray, gray to brown in the center, reaching 46–50 mm diam in 7 days at 25 °C. Colonies on MEA flat, covered by white aerial mycelium, white margin and light orange in the center, reaching 30–35 mm diam after 7 days at 25 °C. Colonies on SNA flat, with entire margin, covered by sparse white aerial mycelium, reaching 20 mm diam after 7 days at 25 °C.

##### Specimens examined.

China, Shaanxi Province, Foping County, Dongshan Park, isolated from leaf spot of *Quercusvariabilis* Bl., *Yong Li*, 11 September 2019 (holotype CAF800057; ex-type living culture: CFCC 54457); *Ibid* (living culture: CFCC 57507).

##### Notes.

Four *Colletotrichum* species are presently known to occur on *Quercus* hosts, *viz. C. clidemiae*, *C.gloeosporioides*, *C.karstii* and *C.theobromicola* (Weir et al. 2012; Liu et al. 2021). *Colletotrichumquercicola* can be distinguished from those species based on any of the loci (ITS, *gapdh*, *chs-1*, *act* and *tub2*) and the fusiform conidia. *Colletotrichumquercicola* is a member of the destructivum species complex and near to *C.tanaceti.* Phylogenetically, this species can be distinguished from *C.tanaceti*CBS 132693 by 88 nucleotide differences in concatenated alignment (20/560 in ITS, 14/274 in *act*, 2/280 in *chs-1*, 17/236 in *gapdh*, and 33/525 in *tub2*) (Damm et al. 2014). Morphologically, *C.quercicola*CFCC 54457 conidia are straight to slightly curved, differing from distinctly curved conidia in *C.tanaceti*CBS 132693 (Damm et al. 2014).

**Figure 4.**
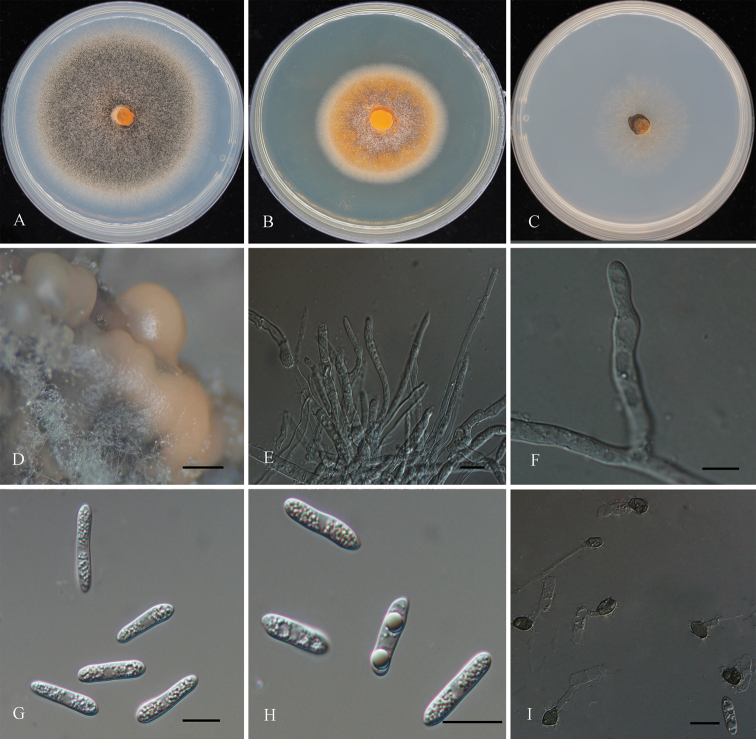
*Colletotrichumquercicola* (CFCC 54457; ex-type) **A** colony on PDA**B** colony on MEA**C** colony on SNA**D** conidiomata formed in PDA**E, F** conidiophores **G, H** conidia **I** appressoria were producing using a slide culture technique **E–H** from PDA. Scale bars: 200 µm (**D**); 50 µm (**E**); 20 µm (**F**); 10 µm (**G–I**).

### ﻿Pathogenicity

**Figure 5.**
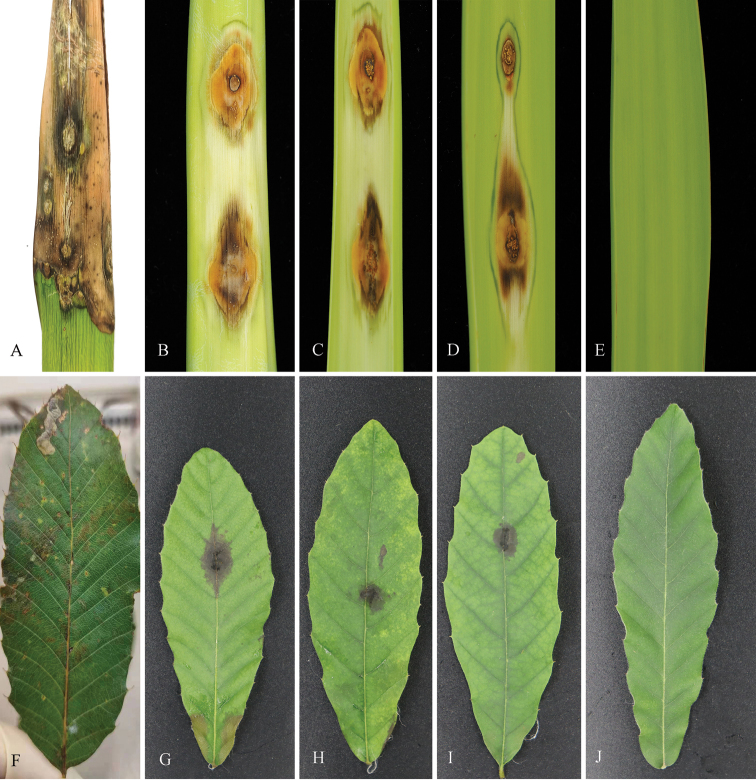
Typical field symptoms of disease and artificial inoculation results **A–E***Yuccagloriosa* leaves **F–J***Quercusvariabilis* leaves **A, F** Anthracnose field symptoms **B–D** Symptoms resulting from *Colletotrichumchinense* (CFCC 57501; ex-type) after 14 days **G–I** symptoms resulting from *Colletotrichumquercicola* (CFCC 54457; ex-type) after 14 days **E, J** symptoms resulting from sterile deionized water after 14 days.

Pathogenicity tests were conducted to confirm Koch’s postulates on *Q.variabilis* leaves for *C.quercicola*, and on *Y.gloriosa* leaves for *C.chinense*. After 14 days of inoculation, necrotic lesions and typical orange conidial masses were observed from the inoculated site of *Y.gloriosa* leaves, and *Q.variabilis* leaves showed brown spot from the inoculated site, whereas all control leaves remained healthy (Fig. 5). Furthermore, *Colletotrichum* isolates could consistently be re-isolated from symptomatic lesions, but never from control leaves. And these isolates were identified as material used for inoculations based on multigene phylogenetic analyses and morphological characters, fulfilling Koch’s postulates.

## ﻿Discussion

In the present study, we collected garden plants with anthracnose symptoms or leaf spots in China. From these samples, the obtained *Colletotrichum* isolates were identified based on morphological features of the asexual morph obtained in culture and five combined loci (ITS, *gapdh*, *chs-1*, *act* and *tub2*) phylogenies. The phylogenetic analyses revealed two novel species, *C.chinense* from *Y.gloriosa* in Beijing, and *C.quercicola* from *Q.variabilis* in the Shaanxi Province, and morphological characters can distinguish these isolates from related species. Pathogenicity test revealed *C.chinense* appearing as a causal agent of *Y.gloriosa* anthracnose and *C.quercicola* as a pathogen of *Q.variabilis* anthracnose.

ITS is evaluated as a universal DNA barcode marker for fungi (Schoch et al. 2012). However, most *Colletotrichum* species could not be distinguished based on ITS only (Cai et al. 2009; Jayawardena et al. 2016). Further, multi-locus DNA sequences, including ITS combined with supplementary barcodes, for which including some of *act*, the intergenic region between DNA lyase and the mating-type (*mat1-2*) gene (*apMat*), DNA lyase (*apn2*), calmodulin (*cal*), *chs-1*, *gapdh*, glutamine synthetase (*gs*), superoxide dismutase (*sod2*) or *tub2* genes for species delimitation (Cannon et al. 2012; Silva et al. 2012a, b; Weir et al. 2012; Vieira et al. 2020). Generally, ITS, *act*, *chs-1*, *gapdh* and *tub2* gene regions have provided adequate resolution to differentiate species within this genus (Bhunjun et al. 2021; Jayawardena et al. 2021; Talhinhas and Baroncelli 2021). In this study, phylogenetic analyses based on five combined loci (ITS, *gapdh*, *chs-1*, *act* and *tub2*) supported that these isolates clustered in a well-supported clade in the agaves and destructivum species complexes with high confidence.

The agaves species complex groups *Colletotrichumagaves*, and four related species, *C.ledebouriae*, *C.neosansevieriae*, *C.euphorbiae* and *C.sansevieriae* (Bhunjun et al. 2021; Talhinhas and Baroncelli 2021). They are unable to be distinguished based on conidial dimensions alone (Table 2). Members of this species complex were assumed to have host specificity (Nakamura et al. 2006; Jayawardena et al. 2021; Talhinhas and Baroncelli 2021). However, three species (*C.ledebouriae*, *C.neosansevieriae* and *C.euphorbiae*) were found only once from its type strain (Crous et al. 2013, 2015, 2016b). Four species, *C.agaves* on *Agave* spp., *C.sansevieriae* on *Sansevieria* sp., *C.ledebouriae* on *Ledebouriafloridunda*, *C.neosansevieriae* on *Sansevieriatrifasciata*, have only been recorded from *Asparagaceae* (Talhinhas and Baroncelli 2021). In this study, *C.chinense* was isolated from symptomatic leaves of *Y.gloriosa*, belonging to the family *Asparagaceae*.

Species in the destructivum species complex are serious pathogens undergoing a hemibiotrophic lifestyle and have been associated with 49 plant species belonging to 41 genera (Damm et al. 2014; Jayawardena et al. 2021; Talhinhas and Baroncelli 2021). Many species appear to have a wide host range, while some species may affect single host species or genera (Damm et al. 2014; Talhinhas and Baroncelli 2021). Typical characteristics of species in this species complex are characterized by the presence of straight or slightly curved conidia with obtuse apices (Bhunjun et al. 2021; Jayawardena et al. 2021). Morphological differences in the size of conidia and appressoria were observed between this species complex (Table 3). The morphological approach alone makes it difficult to distinguish in this complex due to few and variable morphological characteristics.

**Table 3.** Morphological comparison of species in the destructivum species complex.

Species	Type	Media for Conidia morph	Hosts	Distribution	Conidia (µm)	Appressoria (µm)	Reference
*C.americae-borealis*	Holotype	SNA	*Medicagosativa*; *Glycyrrhizauralensis*	America; China	(13.5–)15.5–18(–19) × 3.5–4	(4.5–)6–10.5(–13) × (3.5–)4–7(–10)	Damm et al. (2014)
*C.antirrhinicola*	Holotype	SNA	*Antirrhinummajus*	New Zealand; Japan	(14.5–)15.5–19(–23.5) × (3.5–) 4–4.5(–5)	(9–)9.5–12(–13.5) × (5–)6–8(–10)	Damm et al. (2014)
*C.atractylodicola*	Holotype	PDA	*Atractylodeslancea*	China	13.5–19 × 4–6.5	7.5–14 × 7–10.5	Xu et al. (2018)
*C.bryoniicola*	Holotype	SNA	*genera of Asteraceae*, *Convolvulaceae*, and *Fabaceae*; etc	Netherlands; Italy	(13.5–)15–18.5(–22) × 4–5(–5.5)	(3.5–) 4–10(–18) × (2.5–)3.5–6.5(–7.5)	Damm et al. (2014)
*C.destructivum*	Epitype	SNA	*Trifolium* spp.; *Bletillaochracea*; *Phragmites* sp.; etc	worldwide	(14–)14.5–16.5(–18) × 3.5–4(–4.5)	(6.5–)10–15.5(–20.5) × (4.5–)5–8(–10.5	Damm et al. (2014)
*C.fuscum*	Epitype	SNA	*Digitalis* spp.; *Heracleum* sp.; *Coreopsislanceolata*	Germany; Italy; Netherlands	(16–)16.5–20(–34) × (3.5–)4–4.5(–5.5)	(6–)8.5–14.5(–19) × (6.5–)7–10(–11.5)	Damm et al. (2014)
*C.higginsianum*	Epitype	SNA	*Brassicaceae*; *Campanula* sp.; *Rumexacetosa*	Italy; Japan, Korea; Trinidad; Tobago; America	(17–)19–20.5(–21) × (3–)3.5–4(–4.5)	(5.5–)10–20(–28.5) × (3.5–) 5–9(–12)	Damm et al. (2014)
*C.lentis*	Holotype	SNA	*Lensculinaris*; *Viciasativa*	Canada; China; Romania	(13–)16–20(–26) × 3–4(–5)	(5–)5.5–7.5(–9) × (3.5–)4.5–6(–6.5)	Damm et al. (2014)
*C.lini*	Epitype	SNA	*Linum* sp.; *Nigella* sp.; *Taraxacum* sp.; etc	France; Germany; America; Ireland; Tunisia;Netherlands	(13–)15–18(–22.5) × (3–)3.5–4(–4.5)	(5–)6.5–10(–12.5) × (4–)4.5–6(–7)	Damm et al. (2014)
*C.neorubicola*	Holotype	PDA	*Rubusidaeus*	China	(14.8–)21.5–22.7(–23.5) × (4–)4.9–5.1(–5.6)	(4–)8.2–10.5(–17.5)× (3.6–)5.6–6.8(–11.7)	Liu et al. (2020)
*C.ocimi*	Holotype	SNA	*Ocimumbasilicum*	Italy; Australia	14.5–15.5(–16.5) × (3.5–)4–4.5	(6.5–)7–13(–15.5) × (4–)4.5–7.5(–9)	Damm et al. (2014)
***C.quercicola***	Holotype	PDA	***Quercusvariabilis***	**China**	**(14–)14.5–17.5(–21.5) × (3–)3.3–4.3(–4.7)**	**(5.7–)6.8–9.7(–10) × (3.2–)4–6(–8)**	**This study**
*C.panacicola*			*Panax* sp.	Eastern Asia	17.0–22.1 × 3.4–5.1	14–8	Takimoto (1919)
*C.pleopeltidis*	Holotype	SNA	*Pleopeltis* sp.	South Africa	(15–)19–23(–25) × (5–)5.5(–6)	Not described	Crous et al. (2021)
*C.pisicola*	Holotype	SNA	*Pisum* sp.	America	(11–)15–21(–29.5) × (3–)3.5–4	(5.5–)7–11.5(–13.5) × (4–)4.5–6(–6.5)	Damm et al. (2014)
*C.shisoi*	Holotype	PDA	*Perillafrutescens*	Japan	(15.0–)17–19(–27.0) × (3.0–)4.0(–5.0)	(7.0–)9.0–10.0(–11.0) × (5.0–)7.0–8.0	Gan et al. (2019)
*C.tabacum*	Neotype	SNA	*Nicotiana* spp., *Centellaasiatica*	France; India; Germany; Madagascar; Zimbabwe	(11·5–) 19–20 (–27) × (3–) 5·5–5·8 (–7·6)	(10–) 11·5–12·5 (–14·5) × (6·5–) 8·5–9·5 (–11·5)	Damm et al. (2014)
*C.tanaceti*	Holotype	SNA	*Tanacetumcinerariifolium*	Australia	(13–)14.5–17.5(–19) × (3–)3.5–4(–4.5)	(5–)6.5–12(–14.6)× (3.5–)4.5–7(–10)	Damm et al. (2014)
*C.utrechtense*	Holotype	PDA	*Trifoliumpratense*	Netherlands	17.5–20.5(–23) × 3.5–4(–4.5)	(7–)10–14.5(–15) × (5–)6.5–9.5(–10)	Barimani et al. (2013)
*C.vignae*	Holotype	SNA	*Vignaunguiculata*	Nigeria	(12–)14–17.5(–18.5) × (3–)3.5–4(–4.5)	(4–)4.5–8.5(–12.4)× (3.5–)4–5(–6.5)	Damm et al. (2014)

Although morphological characters may not prove taxonomically informative for species differentiation within species complex, they are considered as a basis to taxonomic segregation for distinguishing species between different species complexes (Cannon et al. 2012; Liu et al. 2022). A polyphasic approach, emphasizing multi-gene phylogenetic analyses combined with analyses of ecological, geographical and morphological data was essential to the identification of *Colletotrichum* species (Cai et al. 2009; Jayawardena et al. 2021; Talhinhas and Baroncelli 2021). In recent years, the classification and species concepts in *Colletotrichum* was changed according to this ideal polyphasic approach (Jayawardena et al. 2021; Talhinhas and Baroncelli 2021; Liu et al. 2022). In the present study, we described two novel species based on molecular sequence analyses and morphological characters, confirming their pathological characterization. To our knowledge, this is the first report of anthracnose on *Y.gloriosa* and *Q.variabilis.* These results may provide an important basis for the prevention and control of this disease.

## Supplementary Material

XML Treatment for Colletotrichum chinense

XML Treatment for Colletotrichum quercicola

## References

[B1] Alizadeh A Javan-Nikkhah M Nourmohammadi Nazarian R Liu F Zare R Fotouhifar KB Stukenbrock EH Damm U (2022) New species of *Colletotrichum* from wild *Poaceae* and *Cyperaceae* plants in Iran. Mycologia 114(1): 89–113. 10.1080/00275514.2021.2008765 35138985

[B2] Barimani M Pethybridge SJ Vaghefi N Hay FS Taylor PWJ (2013) A new anthracnose disease of pyrethrum caused by *Colletotrichumtanaceti* sp. nov. Plant Pathology 62(6): 1248–1257. 10.1111/ppa.12054

[B3] Bhadauria V MacLachlan R Pozniak C Cohen-Skalie A Li L Halliday J Banniza S (2019) Genetic map-guided genome assembly reveals a virulence-governing minichromosome in the lentil anthracnose pathogen *Colletotrichumlentis*. The New Phytologist 221(1): 431–445. 10.1111/nph.15369 PMC6668012 30076781

[B4] Bhunjun CS Phukhamsakda C Jayawardena RS Jeewon R Promputtha I Hyde KD (2021) Investigating species boundaries in *Colletotrichum*. Fungal Diversity 107(1): 107–127. 10.1007/s13225-021-00471-z

[B5] Boratyn GM Camacho C Cooper PS Coulouris G Fong A Ma N Madden TL Matten WT McGinnis SD Merezhuk Y Raytselis Y Sayers EW Tao T Ye J Zaretskaya I (2013) BLAST: A more efficient report with usability improvements. Nucleic Acids Research 41(W1): 29–33. 10.1093/nar/gkt282 PMC3692093 23609542

[B6] Cai L Hyde KD Taylor PWJ Weir BS Waller JM Abang MM Zhang JZ Yang YL Phoulivong S Liu ZY Prihastuti H Shivas RG McKenzie RHC Johnston PR (2009) A polyphasic approach for studying *Colletotrichum*. Fungal Diversity 39: 183–204.

[B7] Cannon PF Damm U Johnston PR Weir BS (2012) *Colletotrichum* – current status and future directions. Studies in Mycology 73: 181–213. 10.3114/sim0014 23136460 PMC3458418

[B8] Carbone I Kohn LM (1999) A method for designing primer sets for speciation studies in filamentous *Ascomycetes*. Mycologia 91(3): 553–556. 10.1080/00275514.1999.12061051

[B9] Corda ACI (1831) Die Pilze Deutschlands In: Sturm J (Ed.) Deutschlands Flora in Abbildungen nach der Natur mit Beschreibungen Sturm, Nürnberg 3(12): 33–64.

[B10] Crous PW Wingfield MJ Guarro J Cheewangkoon R van der Bank M Swart WJ Stchigel AM Cano-Lira JF Roux J Madrid H Damm U Wood AR Shuttleworth LA Hodges CS Munster M de Jesús Yáñez-Morales M Zúñiga-Estrada L Cruywagen EM De Hoog GS Silvera C Najafzadeh J Davison EM Davison PJN Barrett MD Barrett RL Manamgoda DS Minnis AM Kleczewski NM Flory SL Castlebury LA Clay K Hyde KD Maússe-Sitoe SND Chen S Lechat C Hairaud M Lesage-Meessen L Pawłowska J Wilk M Śliwińska-Wyrzychowska A Mętrak M Wrzosek M Pavlic-Zupanc D Maleme HM Slippers B Mac Cormack WP Archuby DI Grünwald NJ Tellería MT Dueñas M Martín MP Marincowitz S de Beer ZW Perez CA Gené J Marin-Felix Y Groenewald JZ (2013) Fungal Planet description sheets: 154–213. Persoonia 31(1): 188–296. 10.3767/003158513X675925 24761043 PMC3904050

[B11] Crous PW Wingfield MJ Guarro J Hernández-Restrepo M Sutton DA Acharya K Barber PA Boekhout T Dimitrov RA Dueñas M Dutta AK Gené J Gouliamova DE Groenewald M Lombard L Morozova OV Sarkar J Smith MT Stchigel AM Wiederhold NP Alexandrova AV Antelmi I Armengol J Barnes I Cano-Lira JF Ruiz RFC Contu M Courtecuisse PR da Silveira AL Decock CA de Goes A Edathodu J Ercole E Firmino AC Fourie A Fournier J Furtado EL Geering ADW Gershenzon J Giraldo A Gramaje D Hammerbacher A He X-L Haryadi D Khemmuk W Kovalenko AE Krawczynski R Laich F Lechat C Lopes UP Madrid H Malysheva EF Marín-Felix Y Martín MP Mostert L Nigro F Pereira OL Picillo B Pinho DB Popov ES Peláez CAR Rooney-Latham S Sandoval-Denis M Shivas RG Silva V Stoilova-Disheva MM Telleria MT Ullah C Unsicker SB van der Merwe NA Vizzini A Wagner H-G Wong PTW Wood AR Groenewald JZ (2015) Fungal Planet description sheets: 320–370. Persoonia 34(1): 167–266. 10.3767/003158515X688433 26240451 PMC4510277

[B12] Crous PW Groenewald JZ Slippers B Wingfield MJ (2016a) Global food and fibre security threatened by current inefficiencies in fungal identification. Philosophical Transactions of the Royal Society B: Biological Sciences 371: 20160024. 10.1098/rstb.2016.0024 PMC5095547 28080994

[B13] Crous PW Wingfield MJ Richardson DM Leroux JJ Strasberg D Edwards J Roets F Hubka V Taylor PWJ Heykoop M Martín MP Moreno G Sutton DA Wiederhold NP Barnes CW Carlavilla JR Gené J Giraldo A Guarnaccia V Guarro J Hernández-Restrepo M Kolařík M Manjón JL Pascoe IG Popov ES Sandoval-Denis M Woudenberg JHC Acharya K Alexandrova AV Alvarado P Barbosa RN Baseia IG Blanchette RA Boekhout T Burgess TI Cano-Lira JF Čmoková A Dimitrov RA Dyakov MY Dueñas M Dutta AK Esteve-Raventós F Fedosova AG Fournier J Gamboa P Gouliamova DE Grebenc T Groenewald M Hanse B Hardy GESTJ Held BW Jurjević Ž Kaewgrajang T Latha KPD Lombard L Luangsa-ard JJ Lysková P Mallátová N Manimohan P Miller AN Mirabolfathy M Morozova OV Obodai M Oliveira NT Ordóñez ME Otto EC Paloi S Peterson SW Phosri C Roux J Salazar WA Sánchez A Sarria GA Shin H-D Silva BDB Silva GA Smith MTH Souza-Motta CM Stchigel AM Stoilova-Disheva MM Sulzbacher MA Telleria MT Toapanta C Traba JM Valenzuela-Lopez N Watling R Groenewald JZ (2016b) Fungal Planet description sheets: 400–468. Persoonia 36(1): 316–458. 10.3767/003158516X692185 27616795 PMC4988374

[B14] Crous PW Hernández-Restrepo M Schumacher RK Cowan DA Maggs-Kölling G Marais E Wingfield MJ Yilmaz N Adan OCG Akulov A Álvarez Duarte E Berraf-Tebal A Bulgakov TS Carnegie AJ de Beer ZW Decock C Dijksterhuis J Duong TA Eichmeier A Hien LT Houbraken JAMP Khanh TN Liem NV Lombard L Lutzoni FM Miadlikowska JM Nel WJ Pascoe IG Roets F Roux J Samson RA Shen M Spetik M Thangavel R Thanh HM Thao LD van Nieuwenhuijzen EJ Zhang JQ Zhang Y Zhao LL Groenewald JZ (2021) New and interesting fungi. 4. Fungal Systematics and Evolution 7(1): 255–343. 10.3114/fuse.2021.07.13 34124627 PMC8165967

[B15] Damm U Woudenberg JHC Cannon PF Crous PW (2009) *Colletotrichum* species with curved conidia from herbaceous hosts. Fungal Diversity 39: 45–87.

[B16] Damm U Cannon PF Woudenberg JHC Johnston PR Weir BS Tan YP Shivas RG Crous PW (2012) The *Colletotrichumboninense* species complex. Studies in Mycology 73: 1–36. 10.3114/sim0002 23136457 PMC3458415

[B17] Damm U O’Connell RJ Groenewald JZ Crous PW (2014) The *Colletotrichumdestructivum* species complex – hemibiotrophic pathogens of forage and field crops. Studies in Mycology 79(1): 49–84. 10.1016/j.simyco.2014.09.003 25492986 PMC4255528

[B18] Darriba D Taboada GL Doallo R Posada D (2012) jModelTest 2: More models, new heuristics and parallel computing. Nature Methods 9(8): 772–772. 10.1038/nmeth.2109 PMC4594756 22847109

[B19] De Silva DD Crous PW Ades PK Hyde KD Taylor PWJ (2017) Life styles of *Colletotrichum* species and implications for plant biosecurity. Fungal Biology Reviews 31(3): 155–168. 10.1016/j.fbr.2017.05.001

[B20] Doyle JJ Doyle JL (1990) Isolation of plant DNA from fresh tissue. Focus (San Francisco, Calif.) 12: 13–15.

[B21] Farr DT Aime MC Rossman AY Palm ME (2006) Species of *Colletotrichum* on *Agavaceae*. Mycological Research 110(12): 1395–1408. 10.1016/j.mycres.2006.09.001 17137776

[B22] Gan P Tsushima A Hiroyama R Narusaka M Takano Y Narusaka Y Kawaradani M Damm U Shirasu K (2019) *Colletotrichumshisoi* sp. nov., an anthracnose pathogen of *Perillafrutescens* in Japan: Molecular phylogenetic, morphological and genomic evidence. Scientific Reports 9(1): 13349. 10.1038/s41598-019-50076-5 31527702 PMC6746953

[B23] Glass NL Donaldson GC (1995) Development of primer sets designed for use with the PCR to amplify conserved genes from filamentous ascomycetes. Applied and Environmental Microbiology 61(4): 1323–1330. 10.1128/aem.61.4.1323-1330.1995 7747954 PMC167388

[B24] Guerber JC Liu B Correll JC Johnston PR (2003) Characterization of diversity in *Colletotrichumacutatum* sensu lato by sequence analysis of two gene introns, mtDNA and intron RFLPs, and mating compatibility. Mycologia 95(5): 872–895. 10.1080/15572536.2004.11833047 21148995

[B25] Huang F Chen GQ Hou X Fu SY Cai L Hyde KD Li HY (2013) *Colletotrichum* species associated with cultivated citrus in China. Fungal Diversity 61(1): 61–74. 10.1007/s13225-013-0232-y

[B26] Hyde KD Cai L McKenzie EHC Yang YL Zhang JZ Prihastuti H (2009a) *Colletotrichum*: A catalogue of confusion. Fungal Diversity 39: 1–17.

[B27] Hyde KD Cai L Cannon PF Crouch JA Crous PW Damm U Goodwin PH Chen H Johnston PR Jones EBG Liu ZY McKenzie EHC Moriwaki J Noireung P Pennycook SR Pfenning LH Prihastuti H Sato T Shivas RG Tan YP Taylor PWJ Weir BS Yang YL Zhang JZ (2009b) *Colletotrichum*—Names in current use. Fungal Diversity 39: 147–182.

[B28] Hyde KD Norphanphoun C Maharachchikumbura SSN Bhat DJ Jones EBG Bundhun D Chen YJ Bao DF Boonmee S Calabon MS Chaiwan N Chethana KWT Dai DQ Dayarathne MC Devadatha B Dissanayake AJ Dissanayake LS Doilom M Dong W Fan XL Goonasekara ID Hongsanan S Huang SK Jayawardena RS Jeewon R Karunarathna A Konta S Kumar V Lin CG Liu JK Liu NG Luangsa-ard J Lumyong S Luo ZL Marasinghe DS McKenzie EHC Niego AGT Niranjan M Perera RH Phukhamsakda C Rathnayaka AR Samarakoon MC Samarakoon SMBC Sarma VV Senanayake IC Shang QJ Stadler M Tibpromma S Wanasinghe DN Wei DP Wijayawardene NN Xiao YP Yang J Zeng XY Zhang SN Xiang MM (2020) Refined families of *Sordariomycetes.* Mycosphere : Journal of Fungal Biology 11(1): 305–1059. 10.5943/mycosphere/11/1/7

[B29] Jayawardena RS Hyde KD Damm U Cai L Liu M Li XH Zhang W Zhao WS Yan JY (2016) Notes on currently accepted species of *Colletotrichum.* Mycosphere 7(8): 1192–1260. 10.5943/mycosphere/si/2c/9

[B30] Jayawardena RS Bhunjun CS Hyde KD Gentekaki E Itthayakorn P (2021) *Colletotrichum*: Lifestyles, biology, morpho-species, species complexes and accepted species. Mycosphere 12(1): 519–669. 10.5943/mycosphere/12/1/7

[B31] Katoh K Rozewicki J Yamada KD (2019) MAFFT online service: Multiple sequence alignment, interactive sequence choice and visualization. Briefings in Bioinformatics 20(4): 1160–1166. 10.1093/bib/bbx108 28968734 PMC6781576

[B32] Khodaei S Arzanlou M Torbati M Eghbali S (2019) Novel hosts in the genus *Colletotrichum* and first report of *C.higginsianum* from Iran. Nova Hedwigia 108(3–4): 449–463. 10.1127/nova_hedwigia/2018/0510

[B33] Kumar S Stecher G Tamura K (2016) MEGA7: Molecular Evolutionary Genetics Analysis Version 7.0 for bigger datasets. Molecular Biology and Evolution 33(7): 1870–1874. 10.1093/molbev/msw054 27004904 PMC8210823

[B34] Lima NB Batista MVDA De Morais MA Barbosa MA Michereff SJ Hyde KD Câmara MP (2013) Five *Colletotrichum* species are responsible for mango anthracnose in northeastern Brazil. Fungal Diversity 61(1): 75–88. 10.1007/s13225-013-0237-6

[B35] Liu F Wang M Damm U Crous PW Cai L (2016) Species boundaries in plant pathogenic fungi: A *Colletotrichum* case study. BMC Evolutionary Biology 16: 81. https://doiorg/101186/s12862-016-0649-5 10.1186/s12862-016-0649-5 PMC4832473 27080690

[B36] Liu LP Wang Y Qiu PL Zhang B Zhang L Wang N Li Y Gao J Hsiang T (2020) *Colletotrichumneorubicola* sp. nov., a new leaf anthracnose pathogen of raspberry from northeast China. Mycological Progress 19(9): 947–955. 10.1007/s11557-020-01614-3

[B37] Liu L Zhang YJ Guo LZ Xu LL (2021) First report of *Colletotrichumgloeosporioides* causing leaf spot on *Cyclobalanopsisglauca* in China. Plant Disease 105(10): 3303. 10.1094/pdis-11-20-2374-pdn

[B38] Liu F Ma ZY Hou LW Diao YZ Wu WP Damm U Song S Cai L (2022) Updating species diversity of *Colletotrichum*, with a phylogenomic overview. Studies in Mycology 101(1): 1–56. 10.3114/sim.2022.101.01 36059896 PMC9365046

[B39] Marin-Felix Y Groenewald JZ Cai L Chen Q Marincowitz S Barnes I Bensch K Braun U Camporesi E Damm U de Beer ZW Dissanayake A Edwards J Giraldo A Hernández-Restrepo M Hyde KD Jayawardena RS Lombard L Luangsa-ard J McTaggart AR Rossman AY Sandoval-Denis M Shen M Shivas RG Tan YP van der Linde EJ Wingfield MJ Wood AR Zhang JQ Zhang Y Crous PW (2017) Genera of phytopathogenic fungi: GOPHY 1. Studies in Mycology 86(1): 99–216. 10.1016/j.simyco.2017.04.002 28663602 PMC5486355

[B40] Mu T Zhang Z Liu R Liu S Li Z Zhang X Xia J (2021) Morphological and molecular phylogenetic analyses reveal three species of *Colletotrichum* in Shandong province, China. MycoKeys 85: 57–71. 10.3897/mycokeys.85.75944 34975280 PMC8674231

[B41] Nakamura M Ohzono M Iwai H Arai K (2006) Anthracnose of *Sansevieriatrifasciata* caused by *Colletotrichumsansevieriae* sp. nov. Journal of General Plant Pathology 72(4): 253–256. 10.1007/s10327-006-0280-1

[B42] Nirenberg HI (1976) Untersuchungen über die morphologische und biologische Differenzierung in der Fusarium-Sektion Liseola. Mitteilungen aus der Biologischen Bundesanstalt für Land- und Forstwirtschaft Berlin-Dahlem 169: 1–117.

[B43] O’Donnell K Cigelnik E (1997) Two divergent intragenomic rDNA ITS2 types within a monophyletic lineage of the fungus *Fusarium* are nonorthologous. Molecular Phylogenetics and Evolution 7(1): 103–116. 10.1006/mpev.1996.0376 9007025

[B44] Rai M Agarkar G (2014) Plant–fungal interactions: What triggers the fungi to switch among lifestyles? 42(3): 428–438. 10.3109/1040841X.2014.958052 25383649

[B45] Réblová M Miller AN Rossman AY Seifert KA Crous PW Hawksworth DL Abdel-Wahab MA Cannon PF Daranagama DA De Beer ZW Huang SK Hyde KD Jayawardena R Jaklitsch W Gareth Jones EB Ju YM Judith C (2016) Recommendations for competing sexual-asexually typified generic names in *Sordariomycetes* (except *Diaporthales*, *Hypocreales*, and *Magnaporthales*). IMA Fungus 7(1): 131–153. 10.5598/imafungus.2016.07.01.08 27433444 PMC4941682

[B46] Ronquist F Teslenko M van der Mark P Ayres DL Darling A Höhna S Larget B Liu L Suchard MA Huelsenbeck JP (2012) MrBayes3.2: Efficient Bayesian phylogenetic inference and model choice across a large model space. Systematic Biology 61(3): 539–542. 10.1093/sysbio/sys029 22357727 PMC3329765

[B47] Schoch CL Seifert KA Huhndorf S Robert V Spouge JL Levesque CA Chen W Bolchacova E Voigt K Crous PW Miller AN Wingfield MJ Aime MC An K-D Bai F-Y Barreto RW Begerow D Bergeron M-J Blackwell M Boekhout T Bogale M Boonyuen N Burgaz AR Buyck B Cai L Cai Q Cardinali G Chaverri P Coppins BJ Crespo A Cubas P Cummings C Damm U de Beer ZW de Hoog GS Del-Prado R Dentinger B Diéguez-Uribeondo J Divakar PK Douglas B Dueñas M Duong TA Eberhardt U Edwards JE Elshahed MS Fliegerova K Furtado M García MA Ge Z-W Griffith GW Griffiths K Groenewald JZ Groenewald M Grube M Gryzenhout M Guo L-D Hagen F Hambleton S Hamelin RC Hansen K Harrold P Heller G Herrera C Hirayama K Hirooka Y Ho H-M Hoffmann K Hofstetter V Högnabba F Hollingsworth PM Hong S-B Hosaka K Houbraken J Hughes K Huhtinen S Hyde KD James T Johnson EM Johnson JE Johnston PR Jones EBG Kelly LJ Kirk PM Knapp DG Kõljalg U Kovács GM Kurtzman CP Landvik S Leavitt SD Liggenstoffer AS Liimatainen K Lombard L Luangsa-ard JJ Lumbsch HT Maganti H Maharachchikumbura SSN Martin MP May TW McTaggart AR Methven AS Meyer W Moncalvo J-M Mongkolsamrit S Nagy LG Nilsson RH Niskanen T Nyilasi I Okada G Okane I Olariaga I Otte J Papp T Park D Petkovits T Pino-Bodas R Quaedvlieg W Raja HA Redecker D Rintoul TL Ruibal C Sarmiento-Ramírez JM Schmitt I Schüßler A Shearer C Sotome K Stefani FOP Stenroos S Stielow B Stockinger H Suetrong S Suh S-O Sung G-H Suzuki M Tanaka K Tedersoo L Telleria MT Tretter E Untereiner WA Urbina H Vágvölgyi C Vialle A Vu TD Walther G Wang Q-M Wang Y Weir BS Weiß M White MM Xu J Yahr R Yang ZL Yurkov A Zamora J-C Zhang N Zhuang W-Y Schindel D (2012) Nuclear ribosomal internal transcribed spacer (ITS) region as a universal DNA barcode marker for fungi. Proceedings of the National Academy of Sciences of the United States of America 109(16): 6241–6246. 10.1073/pnas.1117018109 22454494 PMC3341068

[B48] Seifert KA (2009) Progress towards DNA barcoding of fungi. Molecular Ecology Resources 9(Suppl. 1): 83–89. 10.1111/j.1755-0998.2009.02635.x 21564968

[B49] Silva DN Talhinhas P Várzea V Cai L Paulo OS Batista D (2012a) Application of the *Apn2/Mat* locus to improve the systematics of the *Colletotrichumgloeosporioides* complex: An example from coffee (*Coffea* spp.) hosts. Mycologia 104(2): 396–409. 10.3852/11-145 22086913

[B50] Silva DN Talhinhas P Cai L Manuel L Gichuru EK Loureiro A Várzea V Paulo OS Batista D (2012b) Host-jump drives rapid and recent ecological speciation of the emergent fungal pathogen *Colletotrichumkahawae*. Molecular Ecology 21(11): 2655–2670. 10.1111/j.1365-294X.2012.05557.x 22519519

[B51] Stamatakis A (2014) RAxML version 8: A tool for phylogenetic analysis and post-analysis of large phylogenies. Bioinformatics 30(9): 1312–1313. 10.1093/bioinformatics/btu033 24451623 PMC3998144

[B52] Stoneman B (1898) A comparative study of the development of some anthracnoses. Botanical Gazette Chicago 26(2): 69–120. 10.1086/327721

[B53] Sutton BC (1992) The genus *Glomerella* and its anamorph *Colletotrichum.* In: Bailey JA, Jeger MJ (Eds) *Colletotrichum*: Biology, Pathogenicity, and Control. CAB International, Wallingford, UK, 1–26.

[B54] Takimoto S (1919) Diseases of medicinal plants (3). Bulletin of the Korean Agricultural Society 14: 24–27.

[B55] Talhinhas P Baroncelli R (2021) *Colletotrichum* species and complexes: geographic distribution, host range and conservation status. Fungal Diversity 110: 109–198. 10.1007/s13225-021-00491-9

[B56] Vieira WAS Bezerra PA Silva AC Veloso JS Câmara MPS Doyle VP Vieira WAdS (2020) Optimal markers for the identification of *Colletotrichum* species. Molecular Phylogenetics and Evolution 143: 106694. 10.1016/j.ympev.2019.106694 31786239

[B57] von Arx JA (1957) Die Arten der Gattung *Colletotrichum* Cda. Phytopathologische Zeitschrift 29: 413–468.

[B58] von Schrenk H Spaulding P (1903) The bitter-rot fungus. Science 17(436): 750–751. 10.1126/science.17.436.750.b 17773464

[B59] Weir BS Johnston PR Damm U (2012) The *Colletotrichumgloeosporioides* species complex. Studies in Mycology 73: 115–180. 10.3114/sim0011 23136459 PMC3458417

[B60] White TJ Bruns T Lee S Taylor JW (1990) Amplification and direct sequencing of ribosomal RNA genes for phylogenetics. In: Innis MA Gelfand DH Sninsky JJ White TJ (Eds) PCR Protocols, a Guide to Methods and Applications.Academic Press, New York, 315–322. 10.1016/B978-0-12-372180-8.50042-1

[B61] Xu H Zhou R Fu J Yuan Y Ge X Damm U (2018) *Colletotrichumatractylodicola* sp. nov.: The anthracnose pathogen of *Atractylodeschinensis* in China. Mycological Progress 17(3): 393–402. 10.1007/s11557-017-1359-0

[B62] Zheng H Yu Z Jiang X Fang L Qiao M (2022) Endophytic *Colletotrichum* species from aquatic plants in southwest China. Journal of Fungi 87(1): 1–29. 10.3390/jof8010087 PMC8779291 35050027

